# SERS with Flexible β-CD@AuNP/PTFE Substrates for In Situ Detection and Identification of PAH Residues on Fruit and Vegetable Surfaces Combined with Lightweight Network

**DOI:** 10.3390/foods12163096

**Published:** 2023-08-17

**Authors:** Mengqing Qiu, Le Tang, Jinghong Wang, Qingshan Xu, Shouguo Zheng, Shizhuang Weng

**Affiliations:** 1 Hefei Institutes of Physical Science, Chinese Academy of Sciences, Hefei 230031, China; qmq_study@126.com (M.Q.); qshxu@aiofm.ac.cn (Q.X.); 2 Science Island Branch of Graduate School, University of Science and Technology of China, Hefei 230026, China; 3 National Engineering Research Center for Agro-Ecological Big Data Analysis & Application, Anhui University, Hefei 230601, China; tang_ahut@126.com (L.T.); hongwang321@163.com (J.W.); 4 Anhui Institute of Innovation for Industrial Technology, Hefei 230088, China

**Keywords:** surface-enhanced Raman spectroscopy, flexible substrate, polycyclic aromatic hydrocarbons, in situ detection, deep learning

## Abstract

The detection of polycyclic aromatic hydrocarbons (PAHs) on fruit and vegetable surfaces is important for protecting human health and ensuring food safety. In this study, a method for the in situ detection and identification of PAH residues on fruit and vegetable surfaces was developed using surface-enhanced Raman spectroscopy (SERS) based on a flexible substrate and lightweight deep learning network. The flexible SERS substrate was fabricated by assembling β-cyclodextrin-modified gold nanoparticles (β-CD@AuNPs) on polytetrafluoroethylene (PTFE) film coated with perfluorinated liquid (β-CD@AuNP/PTFE). The concentrations of benzo(a)pyrene (BaP), naphthalene (Nap), and pyrene (Pyr) residues on fruit and vegetable surfaces could be detected at 0.25, 0.5, and 0.25 μg/cm^2^, respectively, and all the relative standard deviations (RSD) were less than 10%, indicating that the β-CD@AuNP/PTFE exhibited high sensitivity and stability. The lightweight network was then used to construct a classification model for identifying various PAH residues. ShuffleNet obtained the best results with accuracies of 100%, 96.61%, and 97.63% for the training, validation, and prediction datasets, respectively. The proposed method realised the in situ detection and identification of various PAH residues on fruit and vegetables with simplicity, celerity, and sensitivity, demonstrating great potential for the rapid, nondestructive analysis of surface contaminant residues in the food-safety field.

## 1. Introduction

Polycyclic aromatic hydrocarbons (PAHs) are persistent organic pollutants that are non-degradable, highly toxic, mutagenic, and carcinogenic [1,2]. Human exposure to PAHs can occur via the inhalation of polluted air, food intake, and skin contact, of which food intake accounts for more than 90% of cases [3,4,5,6]. In particular, fruit and vegetable surfaces tend to attract large deposits of PAHs owing to their long-term exposure to the atmosphere [7,8]. Therefore, it is of great scientific and practical significance to detect PAH residues on fruit and vegetable surfaces because of their strong carcinogenicity and teratogenicity.

In recent years, spectroscopic methods—such as colorimetry, fluorescence spectroscopy, near-infrared spectroscopy, and surface-enhanced Raman spectroscopy (SERS)—have been widely used in PAH analysis because of their efficiency, sensitivity, and automation [9,10,11,12]. Although PAHs exhibit macromolecular fluorescence, conventional fluorescence spectra are easily limited by the broadening of emission bands and it can be difficult to distinguish similar molecules because of their low specificity. SERS is a vibrational spectroscopy technique that provides information on the structural characteristics of molecules, enhances Raman scattering, and has been widely used in fast-trace analysis [13,14,15]. The key to SERS applications is the fabrication of nanostructures with local surface plasmon resonance as SERS-active substrates, and the interaction between the substrates and targets [16]. However, the adsorption of most PAH molecules onto the surface of metal nanoparticles (NPs) is low and resonance Raman scattering does not occur, hindering the effective SERS detection of PAHs. To solve this problem, many researchers have attempted to prepare functionalised plasma nanostructures by modifying the surfactants, antigens, antibodies, and supramolecules on the NP surfaces to promote target binding to SERS substrates [17,18]. However, these strategies are subject to interference from functionalised molecules during SERS detection.

Our previous studies [19] showed that β-cyclodextrins (β-CDs) modified on the surface of gold NPs (AuNPs) can effectively trap PAHs to form host–guest compounds because of their hydrophobic inner cavities (which exhibit a cyclooligosaccharide structure); β-CDs also exhibit weak Raman scattering properties that can reduce interference. Moreover, the surfaces of most fruit and vegetables are irregular and uneven. To improve the application of SERS technology for the detection of irregular sample surfaces, many researchers have attempted to construct flexible SERS-active substrates by assembling metal NPs on flexible materials—such as polymethylmethacrylate (PMMA), tape, and poly(ethylene terephthalate) (PET), which can be easily wrapped or formed to collect analytes from irregular sample surfaces [20,21,22]. Although these methods could achieve in situ detection, their sensitivity and stability required further optimisation because of the viscosity of some of the flexible materials that destroyed nanoarray structures during the stripping process. The superhydrophobic film could effectively narrow the gap between NPs under the action of hydrophobicity to generate a large number of hot spots, which could enhance the SERS signal, and was an effective method for introducing molecules into hot spot regions [23,24]. However, most reported superhydrophobic SERS substrates required various nanofabrication techniques—such as electron beam lithography, optical lithography, and reactive ion etching—thus increasing the cost of actual SERS applications [25,26]. Inexpensive polytetrafluoroethylene (PTFE) films, with the advantage of having a low surface tension, could be combined with lubricants to prepare flexible and hydrophobic platforms that contributed to the generation of hot spots and eliminated the effects of coffee rings and viscosity [27]. Consequently, PTFE films exhibited strong practical application potential for the in situ, sensitive, and stable SERS detection of PAH residues on the irregular surfaces of fruits and vegetables.

To achieve rapid, intelligent, and automated analysis, SERS spectra can be combined with deep learning (DL) methods to build a determination model [28,29]. In particular, lightweight DL networks—such as SqueezeNet, Xception, MobileNet, and ShuffleNet developed based on the representative convolutional neural network (CNN)—have been widely used because of small parameters, low computational overhead, and high precision, showing a higher specificity and sensitivity compared to typical chemometric analysis [30,31,32,33]. For example, Weng et al. [34] used SqueezeNet to develop regression models for the analysis of chlormequat chloride and acephate; excellent performance was obtained with coefficients of determination (*R*^2^) of 0.9836 and 0.9826 and root-mean-square errors (RMSEs) of 0.49 and 4.08, respectively. Wang et al. [35] proposed a novel regression model, a lightweight one-dimensional CNN, for predicting the nicotine content in tobacco leaves with *R*^2^ and RMSE values of 0.95 and 0.14, respectively. These results demonstrated that lightweight networks were suitable for the rapid, accurate analysis of SERS spectra. Consequently, SqueezeNet, MobileNet, and ShuffleNet were used to build classification models for the analysis of various PAH residues on fruit and vegetable surfaces.

**Figure 1:**
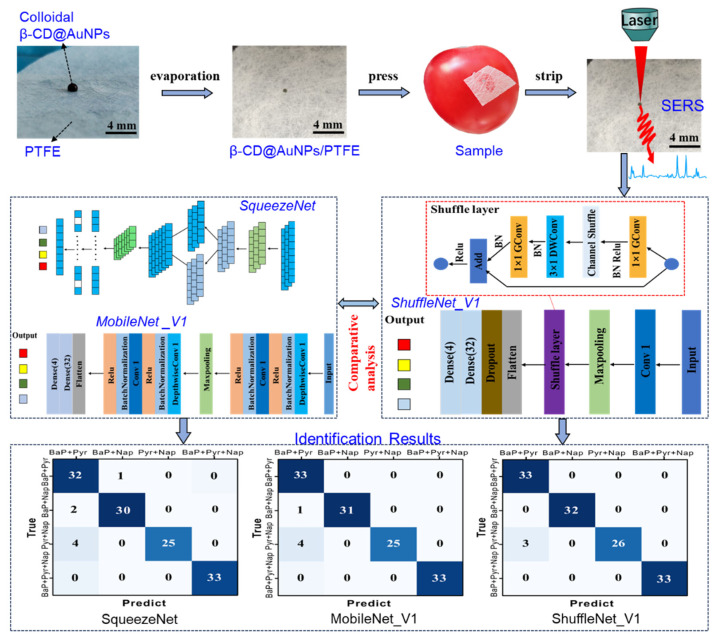
Schematic diagram of the flexible β-CD@AuNP/PTFE combined with lightweight networks to detect PAH residues on fruit and vegetable surfaces.

In summary, this study aims to develop a method for the in situ detection and identification of various PAH residues on fruit and vegetable surfaces using flexible β-CD@AuNP/PTFE substrates and lightweight DL networks (Figure 1). The β-CD@AuNP/PTFE was prepared by assembling β-CD@AuNPs on a flexible PTFE film coated with perfluorinated liquid and the PAHs were detected based on the flexible substrate. SqueezeNet, MobileNet, and ShuffleNet were used to construct an intelligent analysis model combined with SERS spectra to classify various PAH residues on the fruit and vegetable surfaces.

## 2. Materials and Methods

### 2.1. Materials

HAuCl_4_·3H_2_O (99%), β-CD (97%), BaP (96%), Pyr (98%), and Nap (99%) were purchased from Sigma-Aldrich. Na_2_HPO_4_, NaCl, acetone, methanol, and cyclohexane were obtained from Sinopharm Chemical Reagent Co., Ltd.(Shanghai, China), PTFE (pore size 0.1 μm, thickness 70 μm) film was bought from Whatman and polydimethylsiloxane (PDMS) was acquired from Anhui Zhongke Material Co., Ltd.(Hefei, China). Adhesive tape, apples, tomatoes, peaches, and cucumbers were purchased from local supermarkets. Ultrapure water (18.25 MΩ) was used in all experiments.

### 2.2. Preparation of Flexible SERS Substrate

Synthesis of β-CD@AuNPs: β-CD@AuNP sol was prepared using β-CD as a reducing agent and stabiliser, according to Zhao et al. [36]. In brief, 5 mL of 0.1 M phosphate buffer (PB), 1 mL of 0.01 M chlorauric acid solvent, 10 mL of 0.01 M β-CD solution were successively added to 35 mL ultrapure water and stirred vigorously until fully mixed. The mixture was heated to a set boiling temperature and maintained at this temperature for 60 min. The scanning electron microscopy (SEM) images (Figure S1 in the Supporting Information) of the prepared β-CD@AuNPs showed that the particle size was uniform at approximately 20–25 nm.

Preparation of β-CD@AuNP/PTFE: The detailed steps for assembling β-CD@AuNPs on a hydrophobic smooth PTFE film were as follows. First, the PTFE film was glued to a 5 × 2 cm glass slide with double-sided adhesive. The slide was then adsorbed on the machine and 0.45 mL of a perfluorinated liquid was dispersed by spin coating at a low speed of 600 rpm for 30 s and a high speed of 1500 rpm for 1 min. The coated film was heated for 30 min to obtain a spare film. Finally, the concentrated 10 uL β-CD@AuNP colloidal solution was poured onto a hydrophobic PTFE film. During the drying process, the contact line shrank because of the low surface friction of the PTFE film. Eventually, the initial droplet was concentrated in the cell domain with a diameter of 0.5–1 mm (Figure S2 in the Supporting Information).

Preparation of β-CD@AuNP/tape: A two-dimensional NP array of β-CD@AuNPs was obtained using a simple liquid–liquid interface self-assembly method [19] and was subsequently transferred onto silicon wafers. A piece of adhesive tape (clipped using scissors) was then used to cover the nanoarray of β-CD@AuNPs, being pressed firmly for 3–5 s. The tape was gently peeled off from the surface of the silicon wafer and β-CD@AuNP/tape was formed by transferring the β-CD@AuNP array onto the tape.

Preparation of β-CD@AuNP/PDMS: Similarly, flexible β-CD@AuNP/PDMS was prepared by transferring monolayer NP arrays of β-CD@AuNPs using PDMS films instead of silicon wafers and air-drying at 25–30 °C.

### 2.3. Preparation of SERS Sample

A solution of 100 μg/mL was obtained by dissolving 10 μg BaP, Pyr, and Nap solid powders in a 0.1 L ethanol solution. Standard solutions of BaP, Pyr, and Nap at different concentrations (10, 8, 5, 2.5, 1, 0.5, 0.1, and 0.05 μg/mL) were prepared by diluting the 100 μg/mL solution with ethanol. Twenty samples were prepared at each concentration. A standard solution was then used to evaluate the effect of flexible β-CD@AuNP/PTFE on SERS detection. To simulate the actual environment, the spiked samples were prepared by spraying 10 μL of BaP, Pyr, and Nap standard solutions with different concentrations on a fixed area (1 × 1 cm^2^) of the fruit and vegetable surfaces, after which they were air-dried at 25–30 °C. With 20 samples at each concentration, 5 spectra were collected for each.

The PAH samples comprised four classes—that is, BaP + Pyr, BaP + Nap, Pyr + Nap, and BaP + Pyr + Nap—with 20 samples in each class, covering a concentration range of 10 μg/mL to 0.05 μg/mL. Similarly, the 10 μL sample solution was sprayed onto a fixed area of the fruit and vegetable surfaces before being air-dried, followed by 10 μL ethanol being sprayed onto the fixed area to dissolve and extract various PAHs. Finally, the prepared SERS substrate was pasted onto the fruit and vegetable surfaces, gently pressed and lifted, the process being repeated two to three times to realise peel surface sampling. After sampling, the substrate was placed on a slide for SERS detection. Five spectra were collected for each sample.

### 2.4. Spectral Measurement

The morphology and structure of the substrates were characterised using scanning electron microscope (SEM, Zeiss, LSM 710, Oberkochen, Germany) and transmission electron microscopy (TEM, JEOL, JEM-2100F, Tokyo, Japan). The SERS signal of the sample was measured using a portable Raman spectrometer (BWTEK, i-Raman785 Plus, Newark, DE, USA) with a 785 nm He-Ne laser and an excitation light source of 150 mW. The integration time was 10 s, the laser power was 10%, and the spectral range was 300–1800 cm^−1^.

### 2.5. Spectral Analysis Methods

SqueezeNet, MobileNet, and ShuffleNet were used to construct a classification model for the rapid, intelligent identification of various PAHs. SqueezeNet is a lightweight network based on a model-compression strategy [37]. The structure of the SqueezeNet used in this study is shown in Figure S3 in the Supporting Information. The first convolution layer and pooling layer are first used for the initial feature extraction; then, a 1 × 1 convolution layer (squeeze layer) is added, followed by a 1 × 1 convolution and a 3 × 1 convolution extended width (expand layer). The features of the two convolution layers are connected and sent to the flatten, dropout, and dense layers. Notably, the pooling operation of SqueezeNet is delayed, which ensures that a larger feature map is convolved, retaining more feature information, thereby effectively improving network performance. The parameter settings for SqueezeNet are listed in Table S1.

The core idea of MobileNet is to use depthwise separable convolution (DSC) instead of general convolution [38]. The MobileNet structure designed in this study is illustrated in Figure S4 in the Supporting Information. The DSC is implemented using DepthwiseConv and the common 1 × 1 convolution module, both of which are followed by batch normalisation and a rectified linear unit (ReLU) for batch normalisation and nonlinearisation. MobileNet comprises two DSC modules with an additional maximum pooling layer for dimensionality reduction, followed by a flatten layer and two dense layers. The parameter settings for MobileNet are listed in Table S1.

The ShuffleNet network includes group convolution and channel shuffling [39], the structure of which is shown in Figure S5 in the Supporting Information. Initial feature extraction is performed using a common convolution layer and a maximum pooling layer, followed by group convolution and channel shuffle using two shuffle layers. In the shuffle layer, the input is first convolved using a 1 × 1 group convolution, after which the channel shuffle module is used to shuffle the feature graphs of each group. The input is then convolved using a 3 × 1 DepthwiseConv and 1 × 1 group. Finally, the obtained input is added to the initial input to realise group convolution with channel shuffle. After extracting features through the two shuffle layers, the entire network can be completed through the flatten, dropout, and two dense layers. The parameter settings for ShuffleNet are listed in Table S1.

### 2.6. Model Evaluation

There were 400 spectra for the four classes of mixed samples; 30% of the spectra were randomly selected as the prediction dataset, the remaining 70% being divided into training and validation datasets in a 3:1 ratio, which were then used to adjust the network hyperparameters. The accuracy of the training, validation, and prediction datasets (*ACC_T_*, *ACC_V_*, and *ACC_P_*), as well as the *Precision*, *Recall*, and *F*1-*score* of the prediction datasets, were used to evaluate the model performance. *Precision* is the percentage of true positives in all predicted positives; *Recall* is the percentage of predicted true positives in all positives; *F*1-*score* is the weighted harmonic average of *Precision* and *Recall*. The *ACC*, *Precision*, *Recall*, and *F*1-*score* may be conveniently calculated using the following expressions:

(1) $$ACC=\frac{TP+TN}{TN+FP+FN+TP}$$

(2) $$Precision=\frac{TP}{TP+FP}$$

(3) $$Recall=\frac{TP}{TP+FN}$$

(4) $$F1-scores=\frac{Precision\times Recall}{Precision+Recall}$$

where *TP* (resp. *TN*) stands for true positive (resp. negative) and *FP* (resp. *FN*) for false positive (resp. negative).

## 3. Results and Discussion

### 3.1. Influence of Different Flexible Substrates on SERS Activity

**Figure 2:**
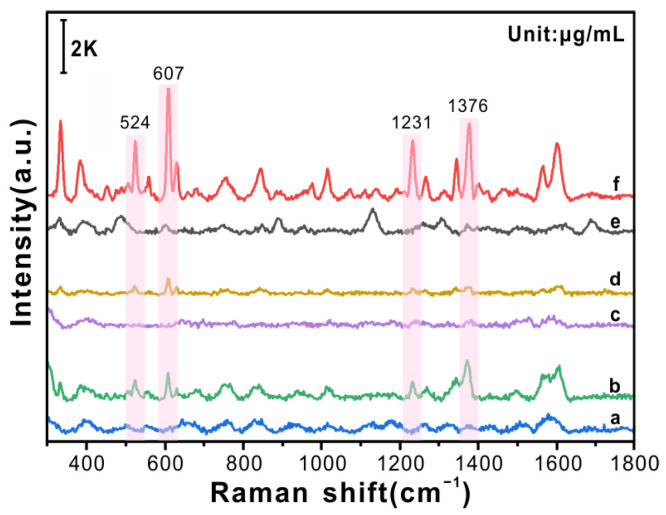
SERS spectra of 10 μg/mL BaP detected based on different flexible substrates: (a) β-CD@AuNP/PDMS, (b) 10 μg/mL BaP detected by β-CD@AuNP/PDMS; (c) β-CD@AuNP/tape, (d) 10 μg/mL BaP detected by β-CD@AuNP/tape; (e) β-CD@AuNP/PTFE, (f) 10 μg/mL BaP detected by β-CD@AuNP/PTFE.

Owing to the interference of several inherent flexible-film characteristics—such as transparency, fluorescent background, viscosity, and impurities—the morphology of the nanoarray structure transferred to its surface was affected, greatly affecting the SERS activity. To select an ideal flexible substrate and realise the in situ detection of trace PAH residues on irregular surfaces in a real-world environment, the influence of three flexible substrates on SERS activity was explored (Figure 2). Figure 2a, c, and e shows the SERS spectra of β-CD@AuNP/PDMS, β-CD@AuNP/tape, and β-CD@AuNP/PTFE, respectively, while the SERS spectra of 10 μg/mL BaP detected by them are shown in Figure 2b, d, and f. As is evident from the figure, β-CD@AuNP/PTFE obtained the best SERS activity (Figure 2f), the characteristic peak of BaP at 524, 607, 1231, and 1376 cm^−1^ being greatly improved. The vibration modes corresponding to each characteristic peak are listed in Table S2 in the Supporting Information. The SERS activity of the β-CD@AuNP/tape is the weakest (Figure 2d), owing to the viscosity and surface roughness of the tape, which easily destroys the two-dimensional nanoarray structure of the β-CD@AuNPs. The PDMS surface is not affected by the viscosity, but the PDMS hydrophobicity is lower than that of the PTFE, so the SERS activity of β-CD@AuNP/PDMS (Figure 2b) is also weaker. The surface hydrophobicity of the PTFE film coated with the perfluorinated liquid limits the diffusion of NPs, which is conducive to reducing the gap between the particles and generating a large number of hot spots. This result can be demonstrated using the SEM image of β-CD@AuNP/PTFE in Figure S6 of the Supporting Information, the gap between the aggregated β-CD@AuNPs being less than 10 nm, which generates abundant hot spots, resulting in a large SERS enhancement with Enhancement Factor of 10^6^~10^7^ (Figure S7 in Supporting Information). Therefore, β-CD@AuNP/PTFE was used as the flexible SERS substrate in subsequent experiments.

### 3.2. SERS Detection of PAHs Based on Flexible β-CD@AuNP/PTFE

**Figure 3:**
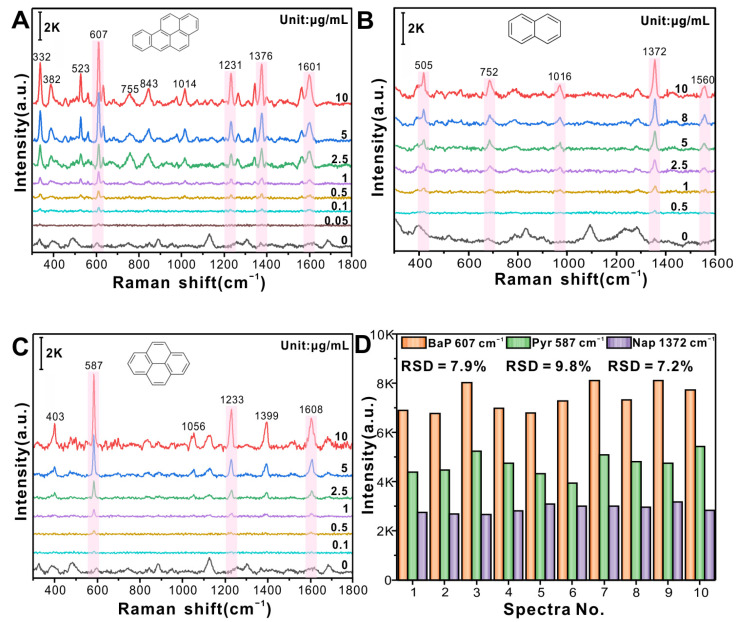
Detection of PAHs by β-CD@AuNP/PTFE; SERS spectra of (**A**) BaP, (**B**) Nap, and (**C**) Pyr standard samples, insets showing their molecular structures; (**D**) RSD values of characteristic peak intensities of 10 μg/mL BaP, Pyr, and Nap at 607, 587, and 1372 cm^−1^, respectively.

Nap, Pyr, and BaP were selected as targets because they are the most representative and widely distributed of the two-, four-, and five-aromatic PAHs, respectively; their structures are shown in Figure 3. SERS detection of BaP, Nap, and Pyr was conducted based on flexible β-CD@AuNP/PTFE, and its sensitivity, reproducibility, and stability were explored. The SERS spectra of BaP, Nap, and Pyr at different concentrations are shown in Figure 3A–C, respectively. The intensities of the characteristic peaks (highlighted in purple) decrease with decreasing concentration. The vibration modes corresponding to the characteristic peaks are listed in Table S2. When the concentration is as low as 0.05, 0.5, and 0.1 μg/mL, BaP at 607 cm^−1^, Nap at 1372 cm^−1^, and Pyr at 587 cm^−1^ still have weak Raman signals, indicating that the flexible β-CD@AuNP/PTFE substrate has strong sensitivity and good universality.

The stability of β-CD@AuNP/PTFE was further evaluated by calculating the relative standard deviation (RSD) of the characteristic peak intensities of the 10 SERS spectra for 10 μg/mL PAHs, as shown in Figure 3D. The RSDs of BaP at 607 cm^−1^, Nap at 1372 cm^−1^, and Pyr at 587 cm^−1^ are 7.9%, 9.8%, and 7.2%, respectively. All RSD values are less than 10%, indicating that the SERS detection of PAHs based on the flexible β-CD@AuNP/PTFE constructed in this study exhibits good stability.

### 3.3. In Situ Detection of PAHs on Fruit and Vegetable Surfaces

**Figure 4:**
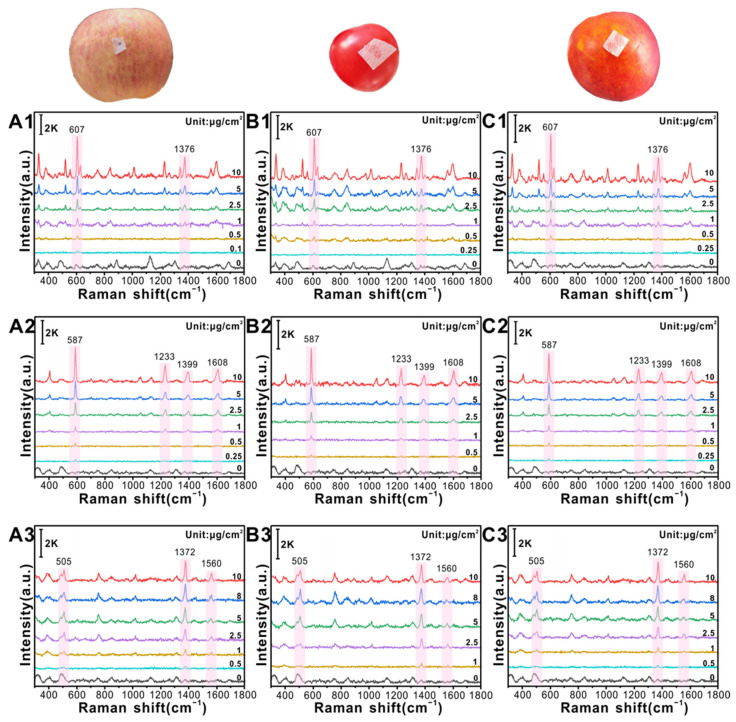
Detection of different concentrations of PAHs on fruit and vegetable surfaces by using a flexible β-CD@AuNP/PTFE substrate. (**A1**–**A3**), (**B1**–**B3**), and (**C1**–**C3**) are the SERS spectra of BaP, Pyr, and Nap with different concentrations on the surface of apple, tomato, and peach, respectively.

The SERS spectra of the spiked samples prepared by spraying BaP, Pyr, and Nap onto the surfaces of apples, tomatoes, and peaches are shown in Figure 4. When BaP concentrations on the surfaces of apples (Figure 4(A1)), tomatoes (Figure 4(B1)) and peaches (Figure 4(C1)) are as low as 0.1, 0.25, and 0.25 μg/cm^2^, respectively, there is still a weak SERS signal at 607 cm^−1^, especially at 0.25 μg/cm^2^, which can be attributed to the C-C and C-H bending vibration modes. The characteristic peaks at 587 cm^−1^ and 1233 cm^−1^ can still be observed at Pyr concentrations as low as 0.25, 0.5, and 0.25 μg/cm^2^ on the surfaces of apples (Figure 4(A2)), tomatoes (Figure 4(B2)), and peaches (Figure 4(C2)), corresponding to C-C stretching and C-H bending vibration modes. The difference in spectral signal intensity between the different peels may be related to the roughness and chemical composition of the peels. When the concentration of Nap on the surface of the apple (Figure 4(A3)), tomato (Figure 4(B3)), and peach (Figure 4(C3)) is as low as 0.5, 1, and 0.5 μg/cm^2^, respectively, the characteristic peak at 1372 cm^−1^ has a weak signal, while the signal at 505 cm^−1^ is almost invisible, which is related to the stretching and bending vibrations of the C-C bond.

Although sensitive SERS detection of Nap on the fruit and vegetable surfaces is realised, the sensitivity is still weaker than that of BaP and Pyr because the C-H bond bending vibration mode of BaP and Pyr has higher polarisability and exhibits stronger peak intensity compared with the C-C bending vibration mode of Nap. The above results indicate that using β-CD@AuNP/PTFE as a flexible SERS substrate can realise the sensitive and in situ detection of PAH residues on the surface of fruits and vegetables, which is of great significance for food safety assessment.

### 3.4. Identification of Various PAHs on Fruit and Vegetable Surfaces

Because there is more than one PAH residue on the surface of fruits and vegetables in the real environment, the main purpose of this work is to realise the identification of various PAHs, without considering a single class. And the competitive adsorption of SERS can also lead to different contributions of PAHs to the SERS signal at the same concentration and can even cover the signals of other PAHs, resulting in low efficiency and accuracy in identifying various PAHs through the manual analysis of spectra. Consequently, in this study, a lightweight network combined with the SERS spectra of various PAHs was used to construct a classification model for intelligent, accurate identification.

**Figure 5:**
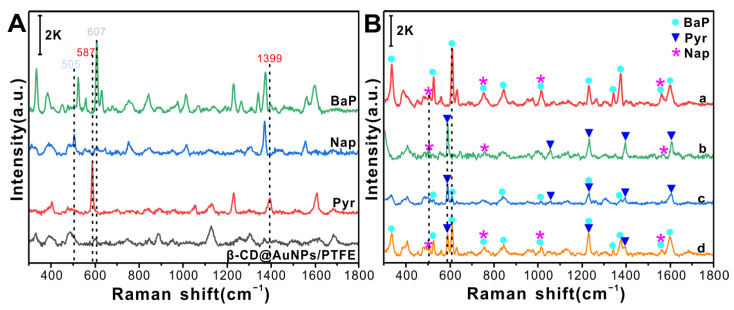
SERS spectra of BaP, Nap, and Pyr at a concentration of 10 μg/mL (**A**); SERS spectra of various PAHs (**B**), from top to bottom: (a) BaP + Nap, (b) Pyr + Nap, (c) BaP + Pyr, (d) BaP + Nap + Pyr.

The SERS spectra of various PAHs were obtained using the flexible β-CD@AuNP/PTFE substrate, as shown in Figure 5. Figure 5A shows the SERS spectra of Pyr, Nap, and BaP. It is evident that the characteristic peak of Nap at 505 cm^−1^ is different from those of Pyr and BaP, and the characteristic peaks of Pyr at 587 and 1399 cm^−1^ are unique without overlapping. Similarly, the characteristic peak of BaP at 607 cm^−1^ is unique. These three targets have unique characteristic peaks that provide a basis for the identification of subsequent detection.

Figure 5B shows the SERS spectra obtained after mixing Pyr, Nap, and BaP. From spectra d of BaP + Pyr + Nap, it is evident that the number of characteristic peaks is more than those of BaP + Nap, Pyr + Nap, and BaP + Pyr (Figure 5B a, b, c). Therefore, the identification performance for BaP + Pyr + Nap may be higher than that for the other types of spectra in subsequent identifications. Additionally, the spectra of a, b, and c in Figure 5B all contain the characteristic peaks of the two PAHs; moreover, there are many overlapping characteristic peaks that make quick and intuitive manual identification difficult to achieve. Consequently, the combination of the SERS spectra of PAHs with lightweight networks is an effective and robust method for constructing recognition models.

**Figure 6:**
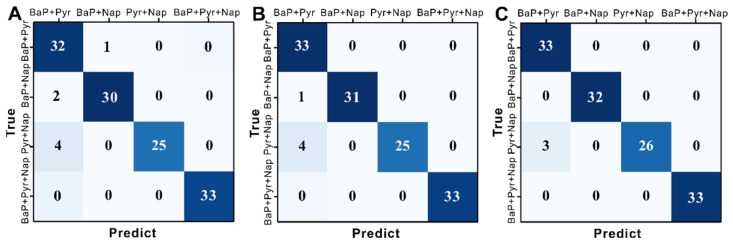
Confusion matrix of (**A**) SqueezeNet, (**B**) MobileNet_V1, and (**C**) ShuffleNet_V1.

The identification results of the SERS spectra of various PAH residues on fruit and vegetable surfaces using the model constructed with three lightweight networks (SqueezeNet, MobileNet, and ShuffleNet) are shown in Table 1. The results obtained from SqueezeNet, with *ACC_T_*, *ACC_V_*, and *ACC_P_* values of 99.57%, 93.22%, and 94.48%, respectively, were unsatisfactory. Based on the *Precision*, *Recall*, and *F*1-*score* of the prediction dataset, SqueezeNet is the best at identifying the mixed spectra of BaP + Pyr + Nap, primarily because of the distinct features of the mixed spectra. But the identification of the BaP + Pyr and Pyr + Nap spectra by this network is poor, indicating that the extracted features are not sufficiently rich, which is consistent with the confusion matrix predicted by the SqueezeNet model (Figure 6A). MobileNet performs better than SqueezeNet, with *ACC_T_*, *ACC_V_*, and *ACC_P_* values of 100%, 94.92%, and 96.06%. The *Precision*, *Recall*, and *F*1-*score* of BaP + Pyr are 100%, 93.94%, and 96.88%, respectively, which are considerably higher than those of SqueezeNet, indicating that MobileNet can effectively capture BaP + Pyr features. Detailed prediction results were obtained from the confusion matrix of the MobileNet model (Figure 6B). Unfortunately, the ability of the network to recognise BaP + Pyr + Nap is low. ShuffleNet achieves the best identification results, with *ACC_T_* = 100%, *ACC_V_* = 96.61%, and *ACC_P_* = 97.63%. These conclusions are also evident from the confusion matrix of the ShuffleNet model shown in Figure 6C, with the identification results of BaP + Pyr + Nap and BaP + Nap by the ShuffleNet model all being correct, and only three Pyr + Nap samples being misclassified as BaP + Pyr. The reason for this result can be found in spectra b and c in Figure 5B, the main characteristic peaks of the spectra of Pyr + Nap and BaP + Pyr being provided by Pyr, and the other weak characteristic peaks from BaP and Nap showing little difference. In general, the results indicate that a lightweight network combined with SERS provides a fast, accurate, and intelligent method for identifying various PAH residues on fruit and vegetable surfaces.

### 3.5. Discussion

In recent years, many studies on in situ detection of targets by using flexible SERS substrates have been widely reported. Alyami et al. [20] fabricated novel AgNP/PDMS composites by self-assembly of organic AgNP solutions on flexible PDMS surfaces; CV and thiram concentrations as low as 1 × 10^−7^ M and 1 × 10^−5^ M were measured on contaminated fish skin and orange peel, respectively. Chen et al. [40] detected three-pesticide residues on tomato peel based on the SERS and flexible tape. Although these methods achieved in situ detection, the sensitivity was low due to the coffee ring effect caused by the weak hydrophobicity of the PDMS and tape surface. Moreover, with the adhesive tape it was easy to destroy the structure of the nanoarray during the “paste and peel off”, resulting in low stability and reproducibility. In this study, we designed the flexible SERS substrate by assembling β-CD@AuNPs on PTFE film coated with perfluorinated liquid, effectively reducing the coffee ring effect and generating a large number of hot spots. The sensitivity and stability of SERS in situ detection were competitive with the strongest results reported by the above work, and the detection process is faster and more convenient, within 1 min.

In addition, DL methods, such as CNNs, recurrent neural networks (RNNs), and generative adversarial networks (GANs), with their strong self-learning ability and excellent fitting ability, were gradually used in spectral analysis to obtain fast and intelligent quantitative or qualitative analysis [28,41]. In particular, CNNs are widely used in the modelling of spectral data by virtue of their advantages with less preprocessing and easy expansion of network architecture. Erzina et al. [42] proposed the advanced route for express and precise recognition of normal and cancer cells by using SERS combined with a CNN, with 100% prediction accuracy. Yu et al. [43] obtained the accurate identification of six representative Vibrio species by combining label-free SERS technology with a CNN, achieving a high accuracy rate of 99.7%. However, these higher accuracy rates were obtained on the basis of building deeper and more complex networks, resulting in an increase in the number of parameters and memory footprint. In this study, the lightweight network developed based on a CNN was used for the first time to construct identification models of various PAHs and the accuracy rate was as high as 97.6%, indicating that this method could improve the computing speed and reduce the memory consumption while ensuring the model accuracy.

## 4. Conclusions

In this study, a flexible SERS substrate of β-CD@AuNP/PTFE combined with a lightweight network was designed to achieve the in situ detection and identification of various PAH residues on fruit and vegetable surfaces. AuNPs were modified with β-CD to enhance adsorption of PAHs. The flexible β-CD@AuNP/PTFE substrate was prepared by assembling β-CD@AuNPs on a PTFE film coated with a perfluorinated solution, contributing to the generation of a large number of hot spots and realising convenient in situ detection. The concentrations of BaP, Pyr, and Nap residues on fruit and vegetable surfaces can still be detected at 0.25, 0.5, and 0.25 μg/cm^2^, and all the RSD values were less than 10%. Subsequently, SqueezeNet, MobileNet, and ShuffleNet networks were used to establish recognition models for various PAH residues on fruit and vegetable surfaces. ShuffleNet obtained the best recognition results with *ACC_T_* = 100%, *ACC_V_* = 96.61%, and *ACC_P_* = 97.63%. These results demonstrated that the proposed method could achieve simple, sensitive, stable, and intelligent in situ detection and identification of various PAH residues on fruit and vegetable surfaces. This method offers great potential for the practical application of rapid, non-destructive analysis of surface contaminant residues in the food industry. However, owing to the large variety and low content of residual PAHs, it is necessary to optimise the SERS substrate to achieve highly sensitive PAH detection in complex matrices. Meanwhile, the SERS spectra of additional types of PAHs should be collected to further improve the recognition performance of the model.

## Figures and Tables

**Table 1:** Identification results of SERS combined with a lightweight network for various PAH residues on fruit and vegetable surfaces.

Methods	Classes	Accuracy (%)	Prediction Dataset		
			*Precision* (%)	*Recall* (%)	*F*1-*Score* (%)
Squeezenet	BaP + Pyr	*ACC_T_* = 99.57 *ACC_V_* = 93.22 *ACC_P_* = 94.48	96.97	84.21	90.14
	BaP + Nap		93.75	96.77	95.24
	Pyr + Nap		86.21	100	92.59
	BaP + Pyr +Nap		100	100	100
Mobilenet_V1	BaP + Pyr	*ACC_T_* = 100 *ACC_V_* = 94.92 *ACC_P_* = 96.06	100	93.94	96.88
	BaP + Nap		96.88	100	98.42
	Pyr + Nap		86.21	100	92.59
	BaP+Pyr+Nap		100	86.84	92.96
Shufflenet_V1	BaP + Pyr	*ACC_T_* = 100 *ACC_V_* = 96.61 *ACC_P_* = 97.63	100	91.67	95.65
	BaP + Nap		100	100	100
	Pyr + Nap		89.66	100	94.55
	BaP + Pyr + Nap		100	100	100

Abbreviations: *ACC*, accuracy of correct classification; *ACC_T_*, *ACC* of the training dataset; *ACC_V_*, *ACC* of the validation dataset; *ACC_P_*, *ACC* of the prediction dataset.

## Data Availability

All related data and methods are presented in this paper. Additional inquiries should be addressed to the corresponding author.

## Supplementary Materials

The following supporting information can be downloaded at: https://www.mdpi.com/article/10.3390/foods12163096/s1. Figure S1: SEM image (A) and Size (B) of the prepared β-CD@AuNPs; Figure S2: The preparation process of β-CD@AuNPs/PTFE; Figure S3: Structure of SqueezeNet; Figure S4: Structure of MobileNet; Figure S5: Structure of ShuffleNet; Figure S6: SEM image of (a) PTFE and (b) β-CD@AuNPs/PTFE; Figure S7: The field distribution of four AuNPs with A particle size of 24 nm with different gaps of (A) 8 nm, (B) 6 nm, (C) 5 nm, (D) 4 nm and (E) 3 nm; (F) Gap distribution of β-CD@AuNPs/PTFE; Table S1: Parameter setting of different models; Table S2: Raman shifts of BaP, Nap and Pyr and the corresponding vibration modes.
