# Supplementary material for: SERS with Flexible β-CD@AuNP/PTFE Substrates for In Situ Detection and Identification of PAH Residues on Fruit and Vegetable Surfaces Combined with Lightweight Network
Source: Foods. 2023 Aug 17;12(16):3096. doi: 10.3390/foods12163096 (PMC10453087; doi:10.3390/foods12163096)
Supplement: Supplementary file 1 [file foods-12-03096-s001.zip › foods-2560291-supplementary.pdf]

SERS with Flexible β-CD@AuNPs/PTFE Substrate for *In-situ* Detection and Identification of PAHs Residues on Fruit and vegetable Surfaces Combined with Lightweight Network

Mengqing Qiu[1,2], Le Tang[3], Jinghong Wang[3], Qingshan Xu[1], Shouguo Zheng[1,4,*],Shizhuang Weng[3,*]

1-Hefei Institutes of Physical Science, Chinese Academy of Sciences, Hefei 230031, People’s Republic of China

2-University of Science and Technology of China, Hefei 230026, People’s Republic of China

3-National Engineering Research Center for Agro-Ecological Big Data Analysis & Application, Anhui University, Hefei 230601, People's Republic of China

4-Anhui Institute of Innovation for Industrial Technology, Hefei 230088, People's Republic of China

Supporting information

SEM image of the prepared β-CD@AuNPs

The SEM image of the prepared β-CD@AuNPs was shown in **Figure S1** A, the particle size is uniform. And the particle size was calculated by using nanomeasure software based on SEM images, as shown in **Figure S1** B, which is about 20-25 nm.

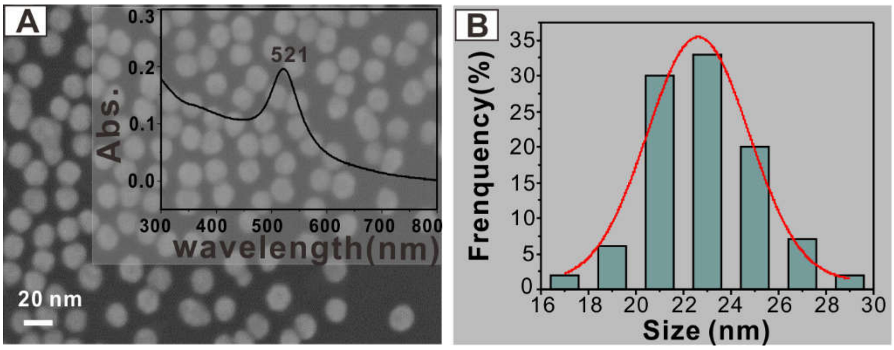

Figure S1. SEM image (A) and Size (B) of the prepared β-CD@AuNPs.

Preparation of β-CD@AuNPs/PTFE

The preparation process of β-CD@AuNPs/PTFE was shown in **Figure S2**. The concentrated 10 uL β-CD@AuNPs colloidal was dropped onto the hydrophobic smooth PTFE film. As the colloidal solution evaporates, the initial droplet can be concentrated into a cell domain with a diameter of 0.5-1 mm.

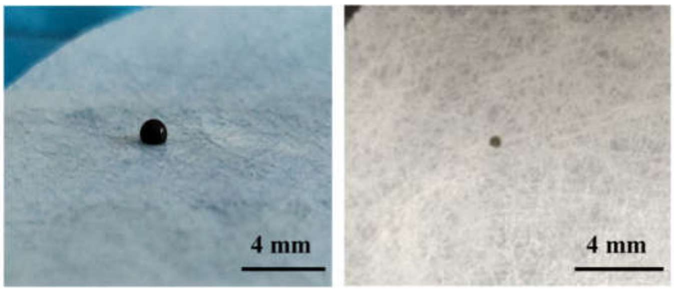

Figure S2. The preparation process of β-CD@AuNPs/PTFE.

The structure of Lightweight deep learning network

The structures of lightweight networks such as SqueezeNet, MobileNet and ShuffleNet used in this study were shown in **Figure S3**, **Figure S4** and **Figure S5**, respectively.

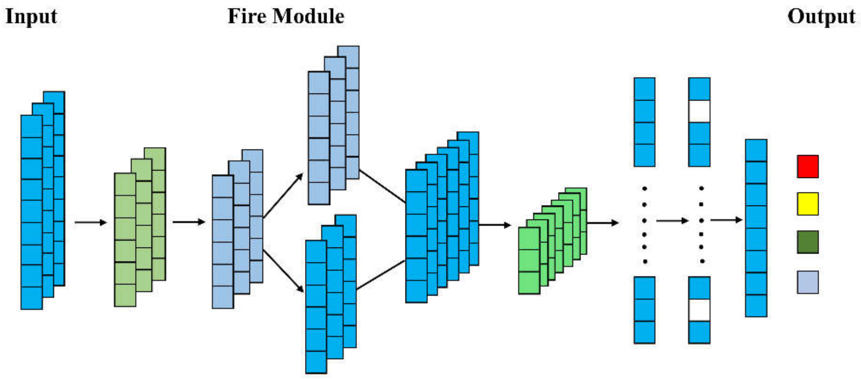

Figure S3. Structure of SqueezeNet.

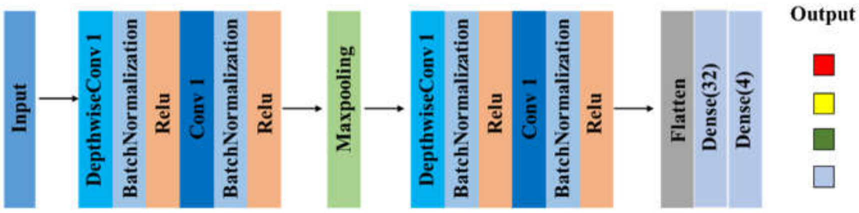

Figure S4. Structure of MobileNet.

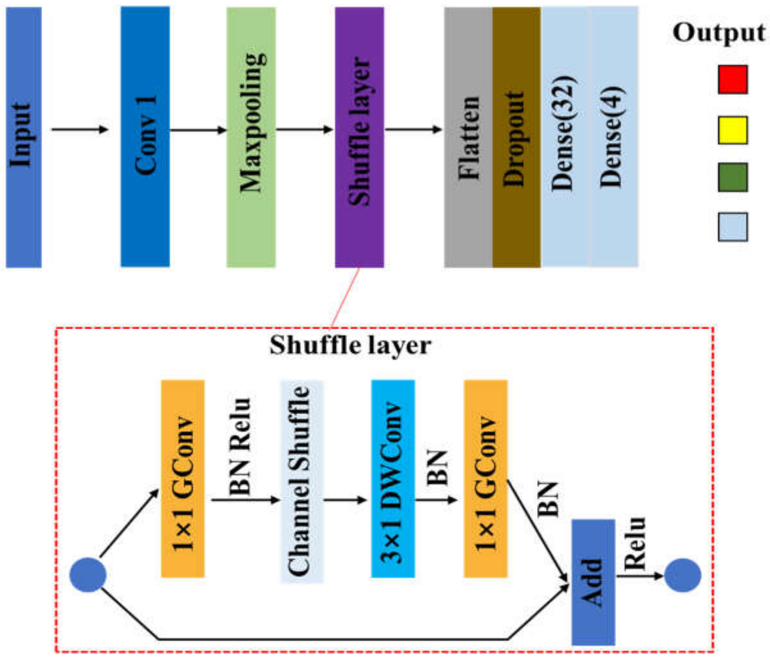

Figure S5. Structure of ShuffleNet.

SEM image of PTFE and β-CD@AuNPs/PTFE

The SEM image of PTFE and β-CD@AuNPs/PTFE were shown in **Figure S6**, the gap between the nanoparticles was less than 10nm, which helps to generate hot spots.

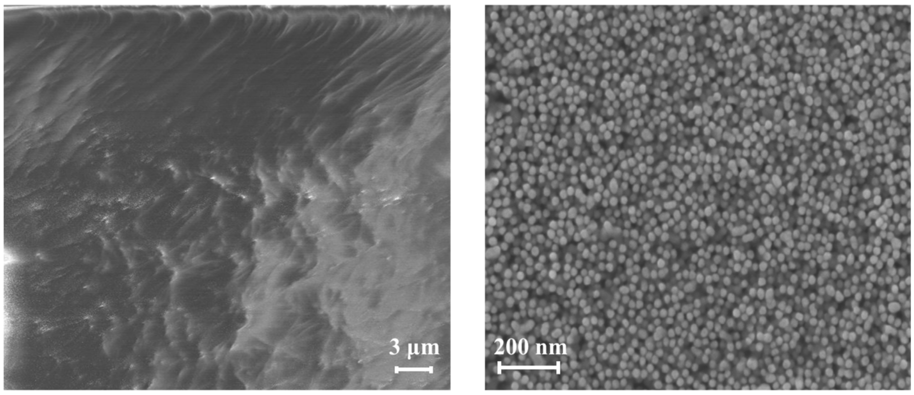

(a) PTFE (b) β-CD@AuNPs/PTFE

Figure S6. SEM image of (a) PTFE and (b) β-CD@AuNPs/PTFE.

The Enhancement Factor of the used SERS platform

The total SERS enhancement comprises two multiplicative contributions, the electromagnetic enhancement (EM) and the chemical enhancement (CE). The enhancement factor of SERS mainly came from the EM, it was proportional to the 4th power of the electromagnetic field intensity. And the effect of chemical enhancement was weak, generally 10^1-10^2. In this study, COMSOL5.6 commercial software package was used to simulate the electromagnetic field intensity of different gaps, and nanomearsurer software was used to calculate that the gap of nanoparticles in the SERS

platform was about 4 nm with $|E/E_0|^2$ of $1.51 * 10^3$, as shown in **Figure S7**. So the Enhancement Factor of the SERS platform was about 10^6~10^7.

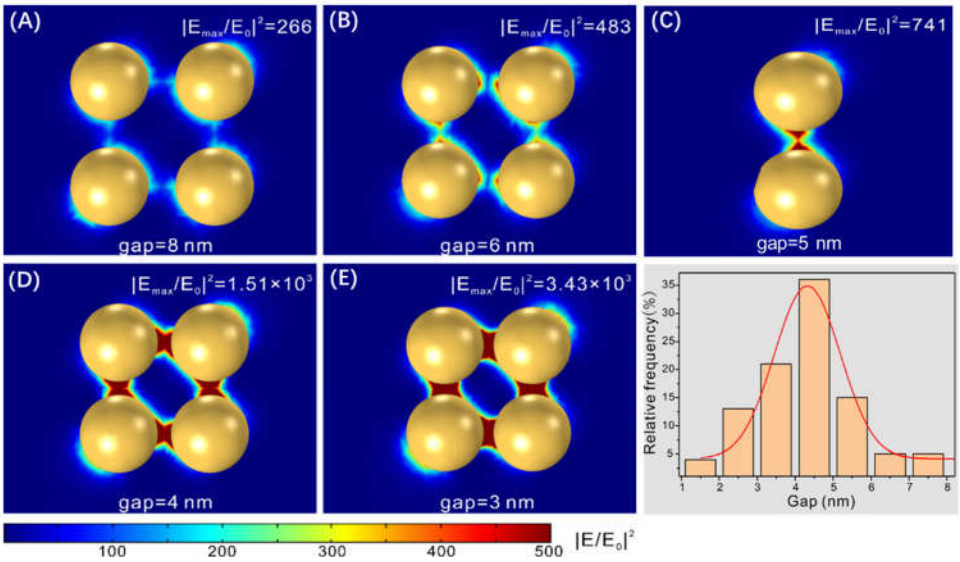

Figure S7. The field distribution of four AuNPs with A particle size of 24 nm with different gaps of (A)8 nm, (B)6 nm, (C) 5 nm, (D) 4 nm and (E) 3 nm; (F) Gap distribution of β-CD@AuNPs/PTFE.

Parameter setting of models

The parameter settings for the Squeezenet, MobileNet, ShuffleNet models are shown in **Table S1**.

Table S1. Parameter setting of different models.

Methods	Parameters
**Squeezenet**	Conv_1 (Relu) 16@3 × 1; Max-pooling_1 2 × 1<br>Conv_2 (Relu) 8@1× 1;<br>Conv_3 (Relu) 12@3 × 1; Conv_4 (Relu) 12@1× 1<br>Concatenate(Conv_3, Conv_4)<br>Conv_5(Relu)8@3×1;<br>Flatten ; dropout(0.5); Dense 32; dense 4<br>optimizer: 'Adam', loss: 'categorical_crossentropy', batch_size=12, epochs=100,lr=0.0001
**MobileNet**	DepthwiseConv_1 5@1×1<br>BatchNormalization_1<br>Activation(Relu)<br>Conv_1 16@1×1<br>BatchNormalization_2<br>Activation(Relu)<br>Max-pooling_1 3×1<br>DepthwiseConv_2 5@1×1<br>BatchNormalization_3<br>Activation(Relu)<br>Conv_2 8@1×1<br>BatchNormalization_4<br>Activation(Relu)<br>Flatten; Dense 32; dense4<br>optimizer: 'Adam', loss: 'categorical _ crossentropy', batch_size=12, epochs=200,lr=0.0001
**ShuffleNet**	Conv_1 (Relu) 16@3 × 1; Max-pooling_1 3 × 1<br>_group_Conv_1(64,(1,1),1,4);<br>BatchNormalization_1; Activation(Relu);<br>_channel_shuffle(activation, 4)<br>DepthwiseConv_1 3×1<br>BatchNormalization_2;<br>_group_conv_2(64,(1,1),1,4);<br>BatchNormalization_3<br>Add_1(Max-pooling_1, BatchNormalization_3)<br>_group_conv_3(64,(1,1),1,4);<br>BatchNormalization_4; Activation(Relu);<br>_channel_shuffle(activation, 4)<br>DepthwiseConv_2 3×1<br>BatchNormalization_5;<br>_group_Conv_4(64,(1,1),1,4);

BatchNormalization_6
Add_2(add_1, BatchNormalization_6)
Flatten; dropout(0.5); Dense 32; dense 4
optimizer: 'Adam', loss: 'categorical_crossentropy', batch_size=12, epochs=100, lr=0.0001

batch_size — the number of training samples sent into the network for each training.

epochs — total number of training sessions for all samples.

lr — learning rate.

The characteristic peak attribution of BaP, Nap and Pyr

The vibration mode corresponding to each obvious characteristic peak is shown in **Table S2**.

Table S2. Raman shifts of BaP, Nap and Pyr and the corresponding vibration modes

PAHs	Raman shift(cm^{-1})	Vibration mode
BaP	332、607	$\delta_{C-C}\delta_{C-H}$
	523	$\delta_{C-C}\delta_{C-H}\tau_{C-C-C-C}$
	843	$\nu_{C-C}\nu_{C-H}$
	1014、1601	$\delta_{C-H}\nu_{C-C}$
	1231	δ_{C-H}
	1376	ν_{C-C-C}
Nap	505	$\nu_{C-C}\delta_{C-C-C}$
	760	ν_{C-H}
	1016、1372、1560	ν_{C-C}
Pyr	403、587	$\nu_{C-C-C-C}$
	1056、1399	$\nu_{C-C}\tau_{C-C-C-H}$
	1233	$\delta_{C-H}\tau_{C-C-C-H}$
	1608	ν_{C-C}

Attention: ν- stretching vibration; δ- bending vibration; τ- twisting vibration
